# Supplementary material for: The G-OBIM tongue model: An accurate open-source biomechanical model of a male human tongue
Source: PLoS Comput Biol. 2025 Sep 8;21(9):e1013378. doi: 10.1371/journal.pcbi.1013378 (PMC12440228; doi:10.1371/journal.pcbi.1013378)
Supplement: S1 Text — (PDF) [file pcbi.1013378.s001.pdf]

Supporting information

S1 Convergence analysis

Fig A plots, for the four points selected in the apical, alveolar, velar and pharyngeal regions, the displacements resulting from the application of the gravity to the tongue model, as a function of mesh density. The displacement error between the reference simulation and the other meshes decreases as the number of nodes increases until it reaches a plateau, indicating convergence.

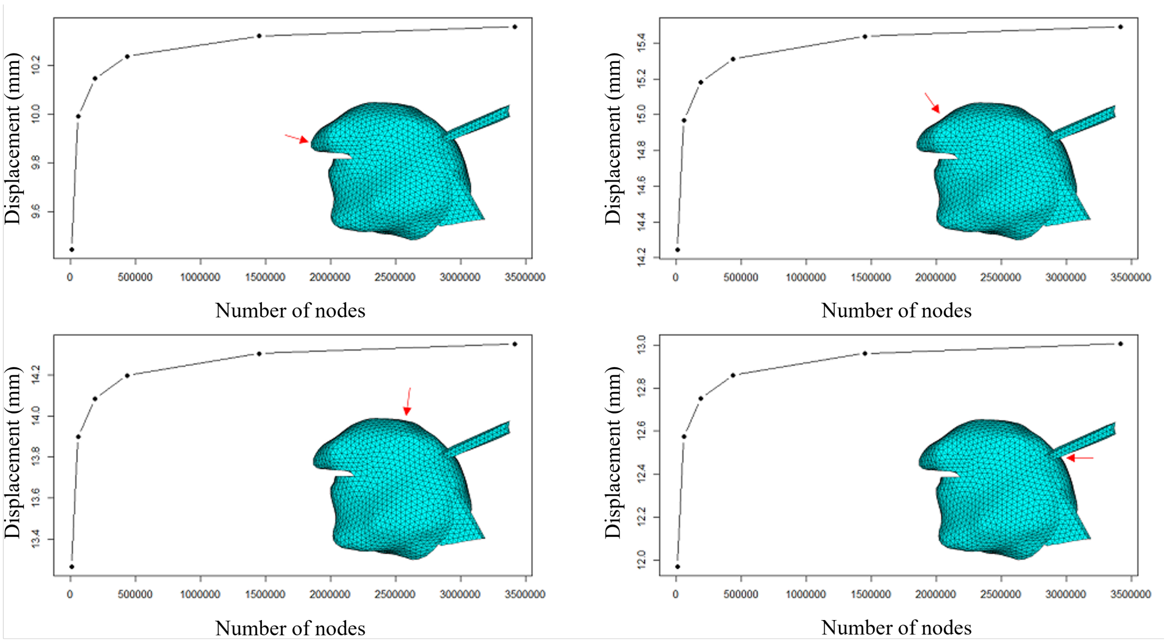

Figure A: Convergence analysis of the tongue mesh. Evolution, as a function of the number of nodes in the mesh, of the amplitude of the displacements of 4 nodes located in key regions of the tongue from the point of view of speech production: apical (top left), alveolar (top right), velar (bottom left), pharyngeal (bottom right).

Table A: Relative error (%) obtained with the different meshes for the considered 4 node positions as compared to the reference simulation with 3412064 nodes.

	**Relative error (%)**			
**Number of nodes**	**Apical**	**Alveolar**	**Velar**	**Pharyngeal**
**7795**	8.8	8.0	7.5	8.0
**57034**	3.6	3.4	3.1	3.3
**187051**	2.0	2.0	1.8	2.0
**436221**	1.2	1.2	1.1	1.1
**1450427**	0.4	0.3	0.3	0.3
**3412064**	Reference			

S2 Elements quality

Table B presents for the reference tongue mesh the mean, minimum, maximum and standard deviation values of four criteria that are commonly used in Finite Element simulations to assess the quality of the elements and, then, the reliability of the simulations: the aspect ratio, the skewness, the orthogonality criteria and the maximum corner angle. Tables C &D give indications about how to rate the quality of the elements based on these four criteria. It can be stated that the huge majority of the tetrahedral elements show excellent characteristics.

Table B: Quality criteria of the elements of the mesh of the tongue (61117 nodes and 41600 tetrahedral elements). The color represents the rating of the element quality based on the nomenclature defined in Tables C &D.

	**Mean**	**Standard deviation**	**Min**	**Max**
**Aspect ratio**	1.952	0.393	1.195	3.983
**Skewness**	0.296	0.115	0.003	0.597
**Orthogonal quality**	0.702	0.114	0.403	0.983
**Maximum corner angle**	98.554°	11.062°	72.708°	135.420°

Table C: Classification table of the qualities of the elements according to two metrics: skewness and orthogonality criteria (Provided in ANSYS documentation).

	Excellent	Very good	Good	Acceptable	Bad	Inacceptable
Skewness	0.0 - 0.25	0.25 - 0.50	0.50 - 0.80	0.80 - 0.94	0.95 - 0.97	0.98 - 1.00
Orthogonal quality	1.00 - 0.95	0.95 - 0.70	0.69 - 0.20	0.20 - 0.15	0.14 - 0.001	0.001 - 0.0

Table D: Classification table of the qualities of the elements according to two metrics: aspect ratio and maximum corner angle (Provided by ANSYS MAPDL software).

	Good	Warning	Error
Aspect ratio	<20	20	100000
Maximum corner angle	<165.0°	165.0°	179.9°

S3 Boundary conditions

S3.1 Fixed nodes

A number of components of the FE meshes are considered to be fixed in the simulations. No displacement is therefore considered for all the nodes associated with these components:

- The maxilla, which belongs to the skull.
- The top ends of the Styloglossus branches are inserted onto the styloid process, which is also part of the skull.
- The lower boundary of the thyroid membrane, which is supposed to be inserted on the thyroid cartilage. Vertical movements of this cartilage do influence tongue positioning via the connection to the hyoid bone via this membrane. At this stage, since we focus on tongue movement with respect to external structures, the thyroid cartilage is not yet modeled, and the lower of the membrane boundary is assumed to be fixed.
- The insertion of the supra- and infra- hyoid muscles, which are also represented by fixed points.

S3.2 Mandible/tongue contacts

The tongue is attached to the mandible by the Genioglossus, Geniohyoid and Mylohyoid muscles. Bonded type contacts (no separation and no sliding between the 2 meshes) between the nodes of the FE meshes of the tongue and those of the mandible, have been defined in the regions concerned by these muscles (Fig B - yellow part). The tongue can also slide over the mandible. These interactions are modeled by sliding contacts (with separation), which we consider to be perfect (Fig B - green part).

S3.3 Hyoid bone/tongue contacts

Via the Hyoglossus muscle, the tongue is directly connected to the greater horn and to the posterior part of the body of the hyoid bone. It is also indirectly connected to the anterior part of the body of this bone through the mouth floor muscles (Geniohyoid and Mylohyoid). These attachment points are also modeled with bonded contacts (Fig C).

S3.4 Maxilla/tongue contacts

The tongue can also come into contact with the palate or the teeth in several cases, such as the pronunciation of the stop consonants /t/ and /k/. These interactions are modeled using sliding contacts (with separation) that we consider as perfect (Fig D).

S3.5 Contacts between the condyle and the mandibular fossa

In the temporomandibular joint region, contacts occur between the condyle of the mandible and the mandibular fossa. This is modeled by perfect sliding contacts with separation [1].

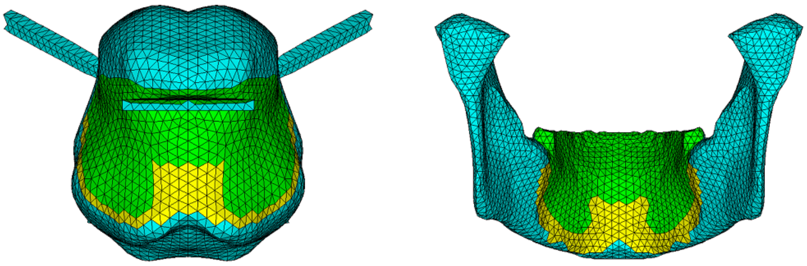

Figure B: Contacts between the mandible and the tongue. Green: Sliding contacts. Yellow: Fixed contacts.

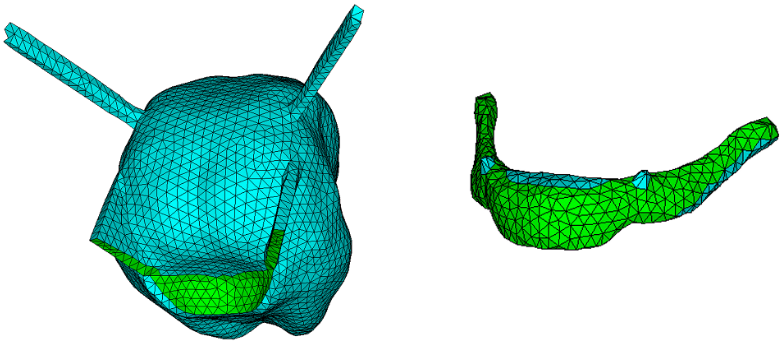

Figure C: Fixed contacts (shown in green) between the hyoid bone and the tongue.

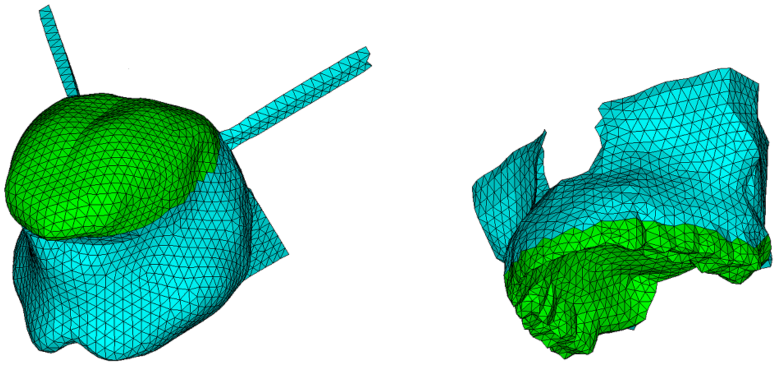

Figure D: Sliding contacts (shown in green) between the maxilla (palate and teeth) and the tongue.

S4 Transient analysis

To study speech production, a proper account of the displacement of the orofacial articulators over time is necessary. The human tongue moves quite fast (10 to 20 cm/s), which implies a significant effect of inertia. Modeling this dynamical behavior requires performing transient FE simulations (simulations resulting from the resolution of the equations of motions). In these simulations the viscous effects are functionally modeled via a Rayleigh damping assuming a damping matrix $C = \alpha_R M + \beta_R K$, where M is the mass matrix and K is the stiffness matrix, with parameters $\alpha_R = 20s^{-1}$ and $\beta_R = 0.0s$.

Piecewise linear activation functions of the tongue muscles have been defined to generate movements between tongue targets. Each piece is defined by its duration and by the activation levels (α) at the start (initial) and target (final) of the movement (Fig E). The activation level α varies between 0 (No constraint) and 1 (Maximal constraint), the maximal constraint level being muscle dependent. In the simulations presented in the paper, the activation evolves within 100ms and the target activation is maintained up to 350ms (in red in Fig E).

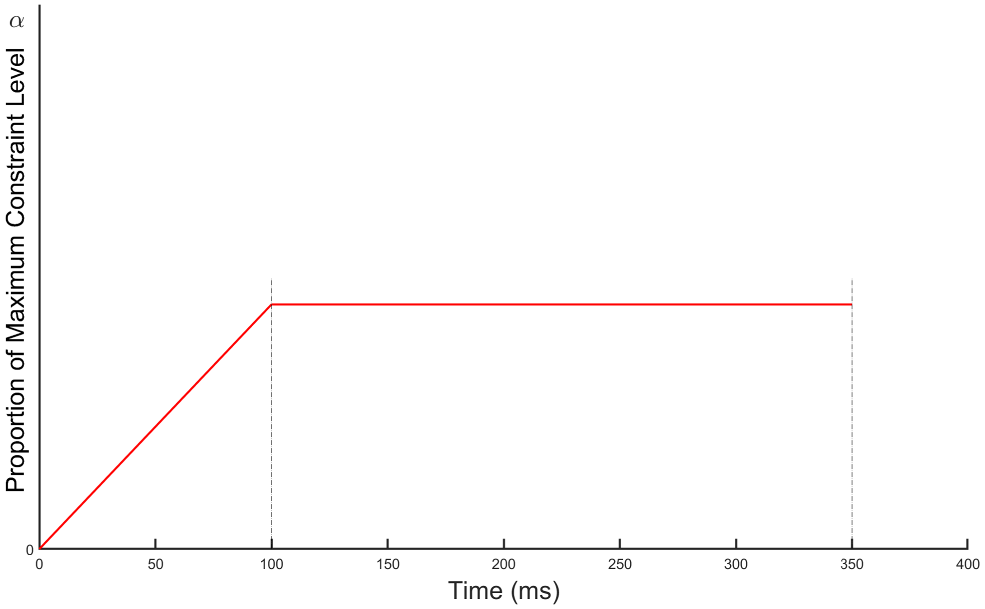

Figure E: Example of a temporal activation sequence. In red, the typical temporal activation pattern used in the simulations whose results are shown in Figs F to Q. These patterns are organized around the notion of transition (increasing or decreasing slope) between target activations (plateaus).

S5 Impact of individual muscles on tongue shape

Figs F to Q represent the displacements (in meters) of the tongue induced by the activation of each tongue muscle separately. Table E provides the values of the active muscle stresses used in these simulations.

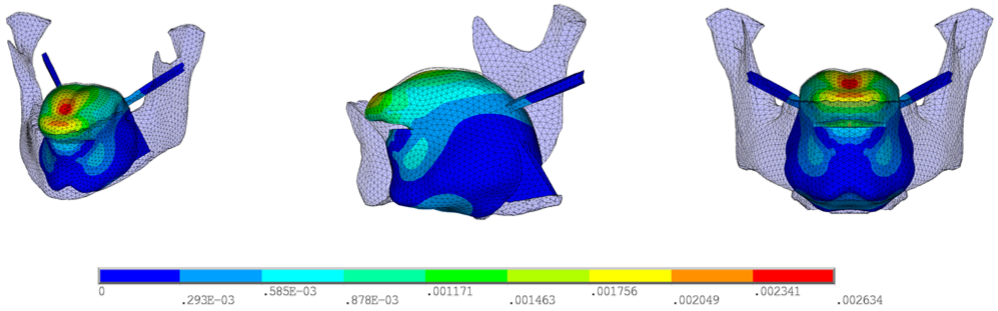

Figure F: Tongue displacements due to the activations of the tongue muscles: Genioglossus anterior (GGa). The colored maps represent the total displacements levels (in meter). The transparent mesh corresponds to the initial configuration of the tongue mesh. Left: cavalier perspective; Center: sagittal or axial view; Right: coronal or axial view.

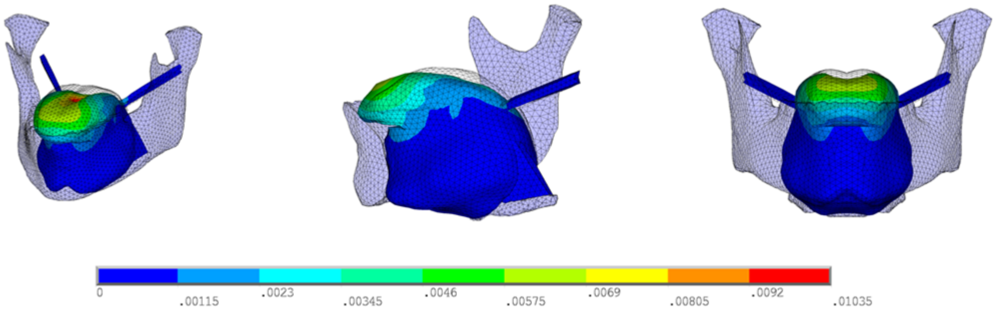

Figure G: Tongue displacements due to the activations of the tongue muscles: Genioglossus medium (GGm). See Fig F for details.

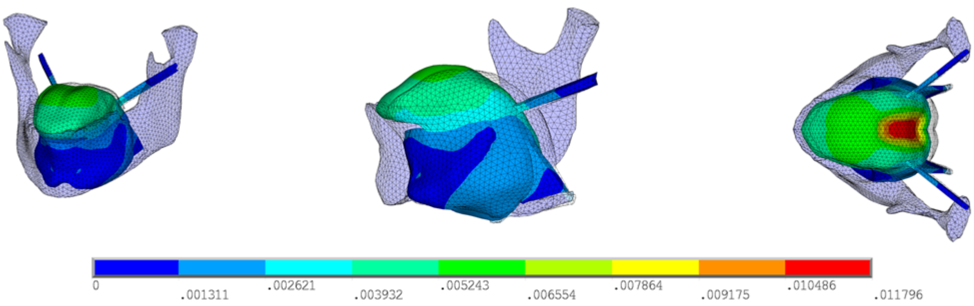

Figure H: Tongue displacements due to the activations of the tongue muscles: Genioglossus posterior (GGp). See Fig F for details.

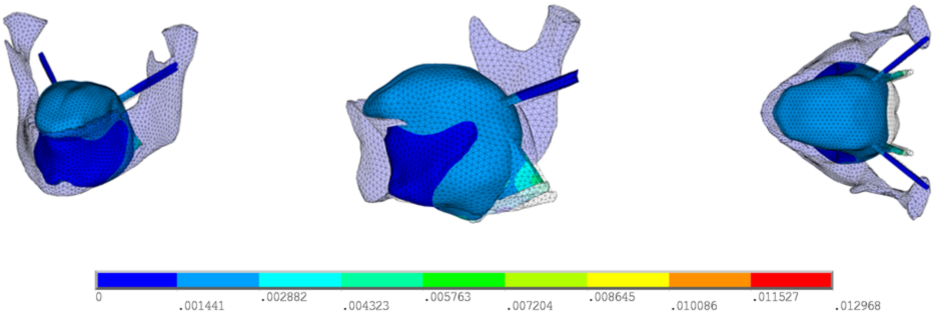

Figure I: Tongue displacements due to the activations of the tongue muscles: Genioglossus horizontal (GGh). See Fig F for details.

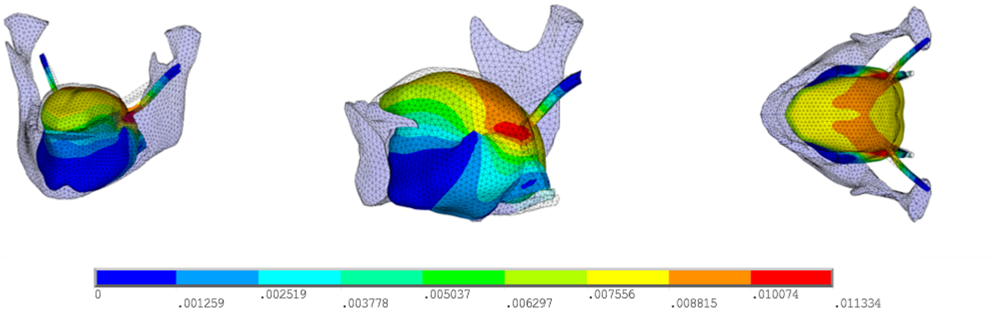

Figure J: Tongue displacements due to the activations of the tongue muscles: Hyoglossus (HG). See Fig F for details.

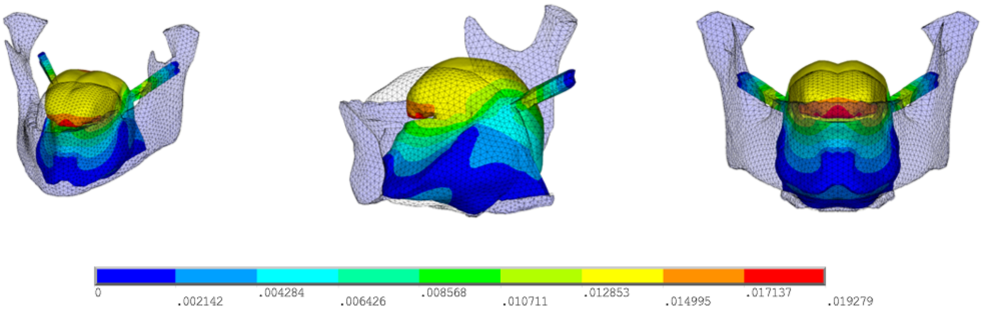

Figure K: Tongue displacements due to the activations of the tongue muscles: Styloglossus (SG). See Fig F for details.

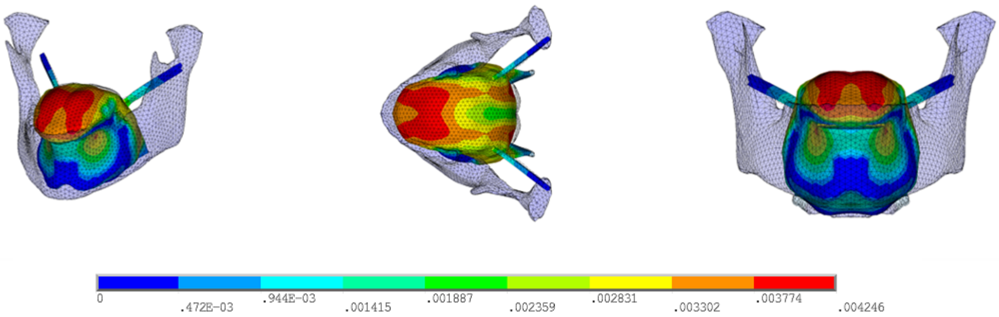

Figure L: Tongue displacements due to the activations of the tongue muscles: Verticalis (Vert). See Fig F for details.

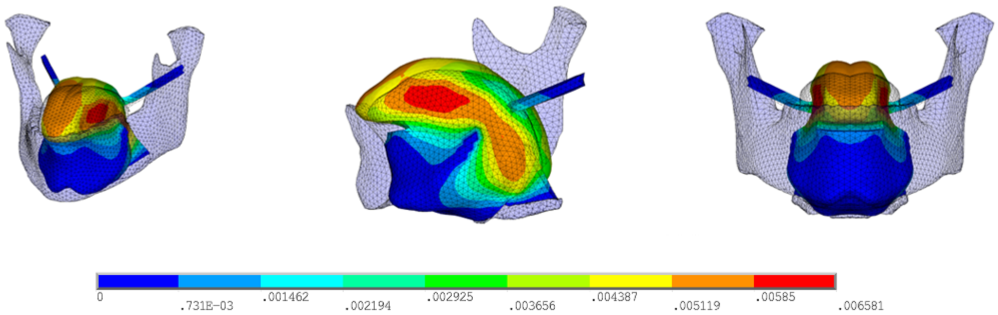

Figure M: Tongue displacements due to the activations of the tongue muscles: Transversalis (Trans). See Fig F for details.

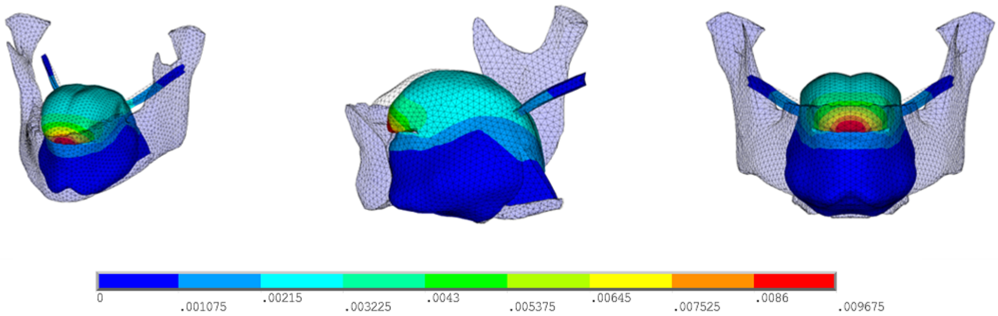

Figure N: Tongue displacements due to the activations of the tongue muscles: Inferior Longitudinalis (IL). See Fig F for details.

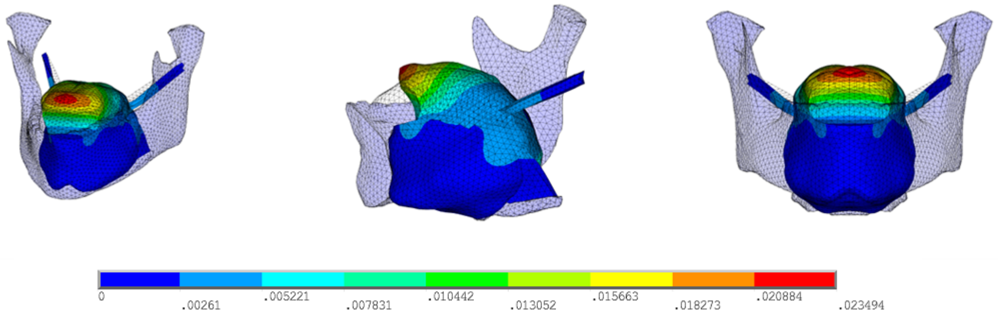

Figure O: Tongue displacements due to the activations of the tongue muscles: Superior Longitudinalis (SL). See Fig F for details.

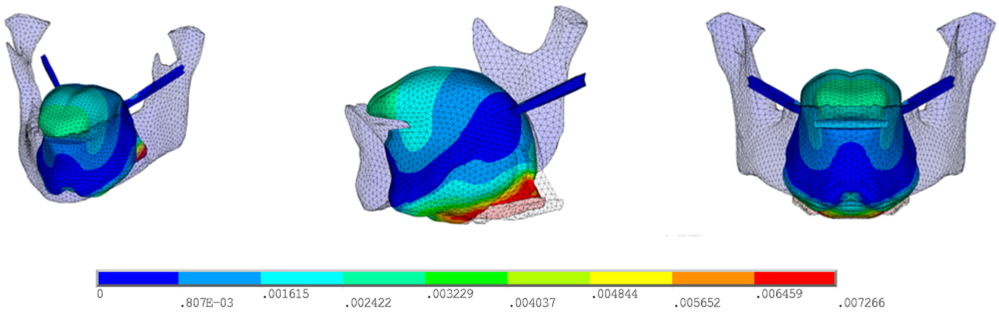

Figure P: Tongue displacements due to the activations of muscles located in the mouth floor: Geniohyoid (GH). The colored maps represent the total displacements levels (in meter). The transparent mesh corresponds to the initial configuration of the tongue mesh. Left: cavalier perspective; Center: sagittal or axial view; Right: coronal or axial view.

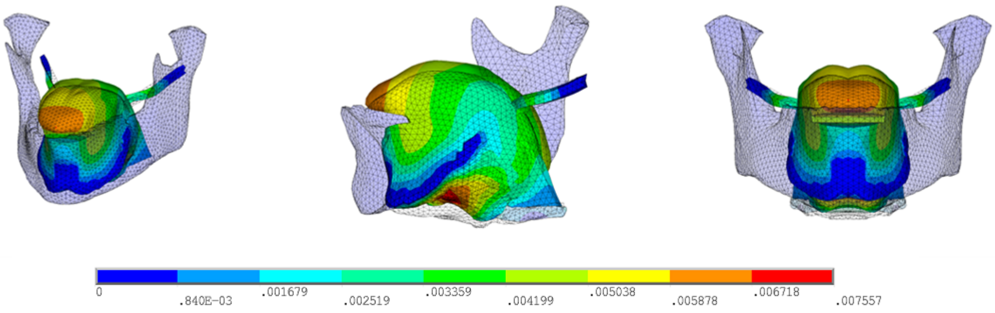

Figure Q: Tongue displacements due to the activations of muscles located in the mouth floor: Mylohyoid (MH). See Fig P for details.

Table E: Active stresses (in Pa) exerted by each tongue muscle in the simulations, whose results are shown in Figs F to Q.

	GGa	GGm	GGp	GGh	SG	HG	Vert	Trans	IL	SL	GH	MH
Active stress (Pa)	5000	10000	4000	10000	150000	5000	1100	1100	10000	4000	25000	20000

The active stress levels of the tongue muscles are in the order of a few kPa (on average $< 10kPa$), except for the Styloglossus muscle. These levels are significantly lower than those proposed by [2], which were probably too high, considering the maximum pressure levels that have been measured when humans were asked to press strongly their tongue against their palate (43 to 78 kPa in healthy adults (see [3] for a review). This is probably due to the fact that the constitutive law used in [2] was too stiff (C_{10} = 1037Pa and C_{20} = 486Pa). In the current model, we use C_{10} = 192Pa and C_{20} = 90Pa, which are the values found experimentally on a fresh cadaver tongue [4] and globally correspond to a stiffness that is 5 times lower in small deformations around the rest position than the stiffness used in [2]. This choice allowed us to have levels of muscle stress that are compatible with the above mentionned measures on humans [3]. In this context, the stress exerted by the Styloglossus (150 kPa) could be considered as too large. However, while the measurements presented in [3] correspond to stresses exerted by the whole tongue against the palate over a surface in the order of 1cm^2, the Styloglossus in the model applies stress via bundles of fibers which cross-sectional areas are in the order of $0.10cm^2$. And in fact, the magnitude of the force generated by the Styloglossus, estimated to 15N, remains within a realistic range.

The independent activation of each functional unit (anterior, medium, posterior, horizontal) of the Genioglossus muscle generates displacements that are in agreement with those described in the literature: lowering of the tongue blade for the anterior Genioglossus, lowering of the tongue dorsum for the medium Genioglossus, compression of the pharyngeal part and elevation of the

tongue surface in the palatal region for the posterior and horizontal Genioglossus. Note that the regions of compression were adapted in the model to account for the deformations observed in vowel /i/ articulated by our reference subject. The Hyoglossus lowers the tongue surface in the palatal region and moves the tongue body backwards. It also slightly increases the size of the subapical cavity and induces an elevation of the greater horn of the hyoid bone. The Styloglossus moves the tongue body towards the back of the vocal tract and raises the tongue dorsum in the velar region. It also moves the tongue tip downwards and backwards, causing the tongue tip to press against the lower part of the anterior surface of the tongue body. The Transversalis narrows the width of the tongue along the transversal (right-to-left) dimension and increases its size in the mid-sagittal plane as a consequence of the conservation of tongue volume due to tissue incompressibility. The Verticalis flattens the tongue in the mid-sagittal plane and generates a slight forward displacement of the tongue surface. The Inferior Longitudinalis essentially moves the tongue tip downwards and backwards. The Superior Longitudinalis efficiently raises the tongue tip towards the palate. Note that this elevation is significantly stronger than the one obtained in the former version of our model [5], which validates the new definition of the apical region, with a subapical cavity (Fig 1, red panel). Note also that the Superior Longitudinalis also slightly compresses the tongue tip. The Geniohyoid moves the posterior part of the tongue forwards and does not contribute to its elevation. It is assumed that it will essentially play a role in maintaining the front-back position of the tongue root. The Mylohyoid raises the mouth floor. Note that the elevation is stronger in its central zone; this is probably related to the absence of connection of the mouth floor with soft tissues in the neck.

References

[1] Koolstra J, Van Eijden T. Combined finite-element and rigid-body analysis of human jaw joint dynamics. Journal of Biomechanics. 2005;38(12):2431–2439.

[2] Buchaillard S, Perrier P, Payan Y. A biomechanical model of cardinal vowel production: Muscle activations and the impact of gravity on tongue positioning. The Journal of the Acoustical Society of America. 2009;126(4):2033–2051.

[3] Adams V, Mathisen B, Baines S, Lazarus C, Callister R. A systematic review and meta-analysis of measurements of tongue and hand strength and endurance using the Iowa Oral Performance Instrument (IOPI). Dysphagia. 2013;28:350–369.

[4] Gerard JM, Ohayon J, Luboz V, Perrier P, Payan Y. Non-linear elastic properties of the lingual and facial tissues assessed by indentation technique: application to the biomechanics of speech production. Medical Engineering & Physics. 2005;27(10):884–892.

[5] Hermant N, Perrier P, Payan Y. Human tongue biomechanical modeling. In: Payan Y, Ohayon J, editors. Biomechanics of Living Organs: Hyperelastic Constitutive Laws for Finite Element Modeling. London, UK: Academic Press; 2017. p. 395–411.

List of Figures

A Convergence analysis of the tongue mesh. Evolution, as a function of the number of nodes in the mesh, of the amplitude of the displacements of 4 nodes located in key regions of the tongue from the point of view of speech production: apical (top left), alveolar (top right), velar (bottom left), pharyngeal (bottom right). . . 1

B Contacts between the mandible and the tongue. Green: Sliding contacts. Yellow: Fixed contacts. 5

C Fixed contacts (shown in green) between the hyoid bone and the tongue. 5

D Sliding contacts (shown in green) between the maxilla (palate and teeth) and the tongue. 5

E Example of a temporal activation sequence. In red, the typical temporal activation pattern used in the simulations whose results are shown in Figs F to Q. These patterns are organized around the notion of transition (increasing or decreasing slope) between target activations (plateaus). 6

F Tongue displacements due to the activations of the tongue muscles: Genioglossus anterior (GGa). The colored maps represent the total displacements levels (in meter). The transparent mesh corresponds to the initial configuration of the tongue mesh. Left: cavalier perspective; Center: sagittal or axial view; Right: coronal or axial view. 7

G Tongue displacements due to the activations of the tongue muscles: Genioglossus medium (GGm). See Fig F for details. 7

H Tongue displacements due to the activations of the tongue muscles: Genioglossus posterior (GGp). See Fig F for details. 7

I Tongue displacements due to the activations of the tongue muscles: Genioglossus horizontal (GGh). See Fig F for details. 8
J Tongue displacements due to the activations of the tongue muscles: Hyoglossus (HG). See Fig F for details. 8
K Tongue displacements due to the activations of the tongue muscles: Styloglossus (SG). See Fig F for details. 8
L Tongue displacements due to the activations of the tongue muscles: Verticalis (Vert). See Fig F for details. 8
M Tongue displacements due to the activations of the tongue muscles: Transversalis (Trans). See Fig F for details. 9
N Tongue displacements due to the activations of the tongue muscles: Inferior Longitudinalis (IL). See Fig F for details. 9
O Tongue displacements due to the activations of the tongue muscles: Superior Longitudinalis (SL). See Fig F for details. 9
P Tongue displacements due to the activations of muscles located in the mouth floor: Geniohyoid (GH). The colored maps represent the total displacements levels (in meter). The transparent mesh corresponds to the initial configuration of the tongue mesh. Left: cavalier perspective; Center: sagittal or axial view; Right: coronal or axial view. 9
Q Tongue displacements due to the activations of muscles located in the mouth floor: Mylohyoid (MH). See Fig P for details. 10

List of Tables

A Relative error (%) obtained with the different meshes for the considered 4 node positions as compared to the reference simulation with 3412064 nodes. 2
B Quality criteria of the elements of the mesh of the tongue (61117 nodes and 41600 tetrahedral elements). The color represents the rating of the element quality based on the nomenclature defined in Tables C &D. 3
C Classification table of the qualities of the elements according to two metrics: skewness and orthogonality criteria (Provided in ANSYS documentation). 3

D Classification table of the qualities of the elements according to two metrics: aspect ratio and maximum corner angle (Provided by ANSYS MAPDL software). 3

E Active stresses (in Pa) exerted by each tongue muscle in the simulations, whose results are shown in Figs F to Q. 10
