# Supplementary material for: The G-OBIM tongue model: An accurate open-source biomechanical model of a male human tongue
Source: PLoS Comput Biol. 2025 Sep 8;21(9):e1013378. doi: 10.1371/journal.pcbi.1013378 (PMC12440228; doi:10.1371/journal.pcbi.1013378)
Supplement: Attachment — Submitted filename: RebuttalLetter.pdf [file pcbi.1013378.s002.pdf]

Rebuttal Letter

Dear Jonghye Woo and Hugues Berry

We would like to thank you and the three reviewers for your interest in our work, and for the helpful and constructive reviews. We really appreciate the time you've invested in that process. We explain below how we took all your suggestions into account, in order to improve the quality of the manuscript.

Sincerely,

Maxime Calka, PhD, on behalf of all the authors.

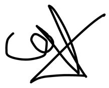

Reviewer #1: The tongue is responsible for major aspects of the production of all vowels, and most consonants, of the world's languages. Having accurate models of it could assist in many subfields of biology, medicine, and phonetics. Several finite-element-type models have appeared in the last several decades, but many of these, especially ones that have been made open-source are of unproven accuracy. The model presented here is impressive due to the care taken to prove convergence, and to make sure that a variety of motor actions observed experimentally with EMA are reproducible by the model. I'm also intrigued by the 4-part functional decomposition of the genioglossus muscle. I don't think enough arguments are provided for the 4 vs. 5 parts, but it's an intriguing and testable proposal. One major confusion I have is why this model is termed open-source. As far as I can tell, it's all performed in ANSYS, which is very expensive software. Is the ANSYS code what the authors plan to make available, or another implementation that could be used by a wider audience? I hope that this is clarified.

Regarding the accessibility of the model, it was indeed developed using ANSYS MAPDL (APDL language), which is a non-free software. However, importantly, the meshes, which describe the tongue geometry and the anatomical muscle placements, are provided in VTK format, allowing compatibility with the huge majority of FE solvers. In addition, the muscle model was developed in Fortran. In the context of our work, it was compiled for use with ANSYS, but again this can be done in other FE solvers too. Boundary conditions are also accessible via the files. Thus, we acknowledge that some work may be needed to use our model in other FE frameworks, and that a direct use will only be possible in the context of ANSYS. However, the most important part of our contribution will be directly available, and the code has been designed to be easily adaptable to other solvers. Additionally, we plan to transfer, in a second stage, this model to open-source platforms such as ArtiSynth (https://www.artisynth.org), as we have done in previous work, if the designers of these platforms are interested.

Reviewer #2: Dear Sir/Mom:
I would to inform you that this paper interesting and fairly well written.
In my opinion, the paper may be suitable for publication in the Journal of PLOS Computational Biology criteria for publication
Yours sincerely,
Dr. Waddhaah M. Alasbahy

Thank you for your comment.

Reviewer #3:

- One of the main contributions of the paper is the partition of the Genioglossus into four functional parts. But it seems that such partitions have been proposed before. Could you please clarify what is the main difference between your approach and those mentioned in e.g., Wrench, A. A. (2024). The compartmental tongue. Journal of Speech, Language, and Hearing Research, 67(10S), 3887-3913.
In the cited paper they also proposed up to 10 divisions of the Genioglossus. Could please also comment on this?

In fact, Wrench, A. A. (2024) proposes a decomposition of the genioglossus into 5 parts. Since the genioglossus muscle is made up in this model of two symmetrical muscles, it corresponds to 10 independent units. From the same perspective, we can say that in our model the Genioglossus includes 8 independent units. In the first submitted version of our manuscript, we cited the work of Harandi et al. (2017), who also proposed a partitioning of the Genioglossus into 5 independent units, and explained why we did not adopt the same approach. Wrench's work is certainly interesting, but from our point of view has significant limitations due to the relative simplicity of the biomechanical model used and the data on tongue shape derived from 2D ultrasound measurements. In our opinion Harandi et al., who used a more realistic model and 3D MRI data, is more relevant.

We modified accordingly the text page 18-19 (lines 293-326):
"Two studies proposed to consider five independently controlled units in the Genioglossus ([53], [54]). Yet, none of them rely on any clear neuro-physiological evidence to support this partitioning. Both studies mentioned the EMG study of Miyawaki et al. [47] who used five electrodes to investigate the spatial distribution of activations in the Genioglossus. However, it is important to remember that this number of electrodes was arbitrarily chosen by Miyawaki et al., in order to demonstrate that "*the genioglossus muscle is, functionally, not "one muscle"*" (page 101). Miyawaki et al. added: "*we do not know how many subdivisions there are in the genioglossus muscle, nor do we know in what manner the subdivisions are voluntarily controlled*" (page 101). In addition, in Miyawaki et al.'s data, the three anterior electrodes showed strong similarities in the time patterns of activations and so did also the two posterior electrodes. In his paper, Wrench mentioned a personal communication with Liancai Mu, who said "*The human GG appears to be composed of at least 4 neuromuscular compartments (NMCs), including GGh and 3 GGo sub-compartments while considering muscle fibre arrangement, fibre type distribution (regionalization), and nerve supply patterns*" ([54], p. 3894). In fact, in order to justify a partitioning of the Genioglossus into more than four parts, Wrench [54] and Harandi et al. [53] looked for the partitioning that enabled the best fitting of tongue shapes observed during speech production. Wrench's work is interesting but its relevance is, in our opinion, limited because it is based on the use of a relatively simple model of the tongue, based on linear elasticity [55]. This simple account of tongue tissue and muscle mechanics is acceptable to describe in a first approximation tongue dynamics. However, it does not seem to be appropriate to simulate tongue deformations with accuracy, since tongue tissue rather behave like hyperelastic materials [32]. Another limitation of Wrench's work lies in the use of 2D views of the tongue recorded with ultrasounds. We know that ultrasounds imaging technique does not provide reliable information on flesh points, but only on the shape of the tongue contour within the beam angle, within which the tongue moves back and forth (e.g. [56]). This does not make this imaging technique a reliable tool for assessing the relationship between anatomical muscle placement and tongue deformations. From this perspective, the work of Harandi et al. [53] is more sophisticated as it used 3D MRI data and more realistic non-linear biomechanical models of the tongue and the jaw. However, in Harandi et al.'s inferred activations of the 5 parts of the Genioglossus (Figure 9 in [53]), a similarity can be observed in the medial parts of the Genioglossus (GGc and GGd) in three of the four speakers. Hence, these results do not convincingly demonstrate the necessity to consider 5 parts in the Genioglossus, and we decided to stick to the decomposition of the oblique fibers of the fan-shaped part of this muscle into 3 functional units, and we obtained satisfactory results."

- Just to clarify. It is mentioned that the stop consonants /t/ and /k/ where capture for ~15 seconds in context. I guess something like /utu/, /iti/... But the durations of these consonants could be between 15-25 ms for /t/ and perhaps ~30 ms for /k/. Could you please clarify this point?

The actual text says: "These sounds were pronounced in isolation and have been hold for 15.328 seconds."

We clarified on page 23, lines 434 – 441 with:
"The MRI acquisition time was 15.3 seconds. The speaker was instructed to artificially sustain the phonemes for that duration (cf. [20] and [21] for more details and validation of this approach). Specifically, for all phonemes, including stops, he was instructed to repeat the utterances first a few times in a natural manner and then to freeze

the consonant or the vowel in the last repetition for the duration of the MRI acquisition. The scan was launched by the operator as soon as he heard that the articulation of the phoneme of interest was being maintained. For instance, for /k/ in /i/ context, the speaker uttered [iki iki iki ik-posture hold for 15s-i]."

- Minor comments:

  - Referencing figures and abbreviations should be consistent. For instance, sometime a figure is referenced as figure 1, Fig 1., Figure 1. The abbreviation Finite Element was defined as FE, but is not used every time.

We have harmonized the « Figure » syntax throughout the text. The FE abbreviation has been harmonized.

- Page 5, Line 111: The parenthesis in "…3D MRI data (240 × 240 × 157 voxels…" is not closed.

The parenthesis is now closed.
