# Supplementary material for: The G-OBIM tongue model: An accurate open-source biomechanical model of a male human tongue
Source: PLoS Comput Biol. 2025 Sep 8;21(9):e1013378. doi: 10.1371/journal.pcbi.1013378 (PMC12440228; doi:10.1371/journal.pcbi.1013378)
Supplement: Attachment — Submitted filename: RebuttalLetter_R3.pdf [file pcbi.1013378.s003.pdf]

Rebuttal Letter

Dear Jonghye Woo and Hugues Berry

We would like to thank you, Khalil Iskarous and the three other reviewers for your interest in our work, and for the helpful and constructive reviews. We really appreciate the time you've invested in that process. We explain below how we took all your suggestions into account, in order to improve the quality of the manuscript.

Sincerely,

Maxime Calka, PhD, on behalf of all the authors.

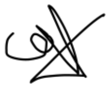

Reviewer #1: I am satisfied with the author's response to my concerns regarding availability, especially their promise to later provide artisynth models. I have Las gone through the responses and changes to my other comments, and those for other reviewers, and I am satisfied with all of the responses and changes.

Thank you for your feedback and for the time you have devoted to our work.

Reviewer #4: The paper itself is interesting and easy to follow for the most part. The new tongue should be a valuable basis for further research.

There are still some shortcomings. For the first part, please note that I am not a native speaker.

We have checked the few specified sentences with ChatGPT and corrected them on this basis.

Some sections of the text are hard to understand. The following sentence [4-7] " Such a high degree of the realism will enable scientists to precisely and quantitatively assess, in a speaker-specific manner, hypotheses about speech motor control and the impact in this respect of tongue anatomy, tongue muscle arrangements and tongue dynamics." for example is grammatically incorrect ("degree of the realism") and generally hard to follow.

We modified this sentence (text page 2, lines 4-7): “Such a high degree of realism will enable scientists to precisely and quantitatively assess, in a speaker-specific manner, hypotheses about speech motor control and the impact of tongue anatomy, muscle arrangements, and tongue dynamics in this context.”

This section from the introduction is also challenging to understand: "The novelty of our approach lies in the fact that we want our new model to be a very faithful representation in morphological and anatomical terms of the tongue of this male individual, so that we can study precisely how this individual controls his tongue during speech production by quantitatively comparing articulatory data collected on this individual during the production of speech sequences and simulations of these same sequences carried out with the model.". While it is understandable, it is also too long and could be split up.

We improved this sentence (text page 4, lines 78-82): “The novelty of our approach lies in the objective of developing a model that faithfully represents the tongue of this male individual in both morphological and anatomical terms. This will allow us to precisely study how he controls his tongue during speech by quantitatively comparing articulatory data collected from this individual with simulations of the same speech sequences performed using the model.”

The abstract still introduces the goal of the paper concisely and mentions many essential aspects of the paper; some metrics and limitations could also be mentioned already.

It is not usual at least in our community to mention in the abstract details such as metrics as well as limitations,

which are typically discussed in the core of the paper.

In the introduction, there is the following passage that I already mentioned above: "The novelty of our approach lies in the fact that we want our new model to be a very faithful representation in morphological and anatomical terms of the tongue of this male individual, so that we can study precisely how this individual controls his tongue during speech production by quantitatively comparing articulatory data collected on this individual during the production of speech sequences and simulations of these same sequences carried out with the model."

I am no expert in this field. Hence, I might be wrong here, but doesn't everyone strive for "a very faithful representation"? It would be better if you would explain what you are doing to achieve a faithful representation instead of mentioning that you are trying to achieve it, in my opinion.

Speech is a cognitive skill that is unique to humans and shared by all the humans. As a consequence, for a long time, speech was studied in a global manner, looking more for invariants shared by all the individuals speaking a given language, essentially disregarding interindividual variability. This was typically the case for speech recognition systems that aimed get rid of the interindividual variability found in the acoustic signal in order to extract the invariants of the language. This was also the case in basic research, such as speech production and speech perception research, where scientists were looking for principles shared by all humans to understand how sounds are articulated in the vocal tract, generated in the acoustic/auditory domain, and finally perceived. Yet, in recent years, a paradigm shift has taken place both in speech technologies with speaker-specific speech synthesizers and in basic research with a trend to account for individual-specific characteristics, such as idiosyncrasies. Our work aligns with this perspective, as we aim to accurately reproduce the tongue shapes of a given specific speaker, in order to evaluate speech motor control hypotheses for this individual, for whom we have a large amount of acoustic and articulatory speech data of various kinds. To better understand this paradigm shift, it is interesting to compare our current work with the earlier study of our group by Buchaillard et al. (2009), which sought to identify general features of vowel production provided thus a qualitative more than a quantitative comparison with speech production data. We are currently working on adapting the model presented in the current manuscript to other specific speakers, using a methodology described in Calka et al., 2023, Alvarez et al., 2024). in order to integrate speaker specific aspects in speech motor control.

In the literature, many studies still rely on generic non-speaker specific models. Examples include the work of Rakotomalala et al (2022), who used a 2D tongue model, or Stavness et al (2012), Gick et al. (2017), Al-Zanoon et al. (2024) or Isazadeh et al. (2024), who all used Buchaillard et al's (2009) 3D model. Our aim is to go beyond this generic approach, in order to provide new insights into motor control issues and their links with individual characteristics.

Regarding the results, I appreciate the thorough analysis of the mesh resolution. I also like your reasoning for selecting the mesh you ultimately chose.

I understand that optimization methods can be costly, but I am still not fond of the reliance on trial and error. The manual adjustments will be difficult to reproduce, and it is also unclear how well this generalizes to other speakers. I still appreciate that you clarified your approach a bit more later on.

We understand your concerns about using a *trial-and-error* approach. However, there is currently no reliable method, based for example on inversion techniques, to perform this work in an automatic and fully reproducible way. Indeed, such methods would require systematic and comprehensive learning of the relationships between muscle activations and tongue shapes in 3D space. For such learning to be accurate, thousands of simulations would be required. This estimate does not come out of the blue. It has its origins in a work carried out in our research group (Rakotomalala et al., 2022) with a much simpler biomechanical model of the tongue (Payan & Perrier, 1997 in its improved version in Perrier et al., 2003). This model is a 2D model, has 221 nodes, is controlled by just 7 muscle commands and implements the linear elasticity of the tissue. Despite this relative simplicity, 25000 simulations were required to satisfactorily learn, with an LSTM, the relationship between the temporal variation of the 7 motor commands and the movements of the 16 nodes that describe the sagittal contour of the tongue. In our 3D hyperelastic model, which has 61117 nodes and involves 12 muscles, we can expect the number of simulations required to be at least 50000. It is important to note that with this model, a simulation of 150ms of speech movements (i.e., the simulation of the production of around 1 phoneme, if we take into account that a typical speech rhythm corresponds to 5 to 7 syllables per second) requires around 3 hours. Under these conditions, it is obvious that the intensive learning required is not feasible, and that no reliable automatic inversion method can then be envisaged. This represents a real challenge for our work, in the aim to use the model in the future for a systematic study of speech motor control and its idiosyncrasies. This is why we are actively working on accelerating simulations, by working on methods for designing a reduced-order, near-real-time model of biomechanics (see our preliminary work in Calka et al., 2021).
We did not state that the muscle activation patterns that we found using this *trial-and-error* approach should generalize to other subjects. On the contrary, we wrote that designing models that implement subject-specific anatomical and morphological characteristics would help discovering subject-specific strategies and idiosyncrasies.

The simulation for the different vowels and consonants is also fascinating. The active stress values also provide great insight. The reasoning behind the muscle activations, combined with the references, is great and easy to follow, especially for people like me who aren't experienced in this kind of work.

If I see it correctly, most assessments made in the results section are visual-based. I think that some additional quantitative results could also be helpful if creating meaningful quantitative results is feasible. This could include error margins or confidence intervals. In terms of generalizability, having a few more reference subjects would also be beneficial, but I also understand that obtaining the necessary data can be very challenging.

We considered the possibility of providing quantitative measures of the similarity between our simulated tongue shapes and the ones produced by our reference speakers. We have not done so for two main reasons.
Firstly, **it is not really straightforward**. Indeed, this requires segmenting the 3D shape of the tongue in the MRI images of our reference speaker, which is difficult to do accurately in regions where the tongue comes into contact with other soft tissues, primarily at the back of the mouth, on the palatal vault and in the pharynx. Such a measurement would then only be possible in the mobile part of the tongue (i.e. in the alveo-palatal and front palatal regions), using a method similar to the one we used in our work where we evaluated our ability to generate a baboon's tongue using a geometric transformation deduced from 3D images of the skull.
(see https://journals.plos.org/ploscompbiol/article?id=10.1371/journal.pcbi.1011808)
Secondly, if a precise segmentation of the tongue were possible, or if we wanted to evaluate the prediction of our model only in the mobile part of the tongue, as we just mentioned above, a quantitative measure of accuracy would be technically possible, but it wouldn't make much scientific sense. Indeed, a human speaker is not capable of reproducing exactly the same tongue posture for a given speech sound from one production to the next, even if he or she wishes to do so. In fact, two repetitions of the same sound under similar conditions will always be different. This is the well-known *token-to-token* variability, which has been widely studied in speech production research, including by some of us (https://www.researchgate.net/publication/233945508_aipuk36_mooshammer). This variability is probably due both to the corruption of motor commands and/or sensory signals by noise and to the fact that somatosensory and auditory feedback are not sufficiently accurate to detect variations below a certain threshold. Therefore, from a scientific point of view, if we want our model to mimic real speech production, we need to evaluate the accuracy of muscle activations that our trial-and-error approach achieves, in the light of the accuracy that a real speaker is capable of achieving in the same process. In other words, the assessment of a quantitative difference between the simulated and the recorded tongue should integrate this natural amount of variability. At this stage we only have a unique experimental image for each sound. Now, the visual comparison that we carried out has some value, since it takes into account at the same time global phonetically crucial characteristics of the tongue shape, such as its anteroposterior positioning and the location and degree of its proximity to the hard palate, and details, such as the position, the depth of the grooving in the alveopalatal region or the position of the highest point of the tongue along its upper contour. While the phonetically crucial characteristics are shared by all speakers in a same language, details reveal speaker-specific strategies and muscle recruitments. This is well-illustrated by the figure below that shows the articulation of the same vowel /i/ in the sagittal plane (front is on the right) by two different native speakers of French: both articulations correspond to a tongue that is protruded and high in the palato-alveolar region, but the tongue contours are quite different depending on the speaker.

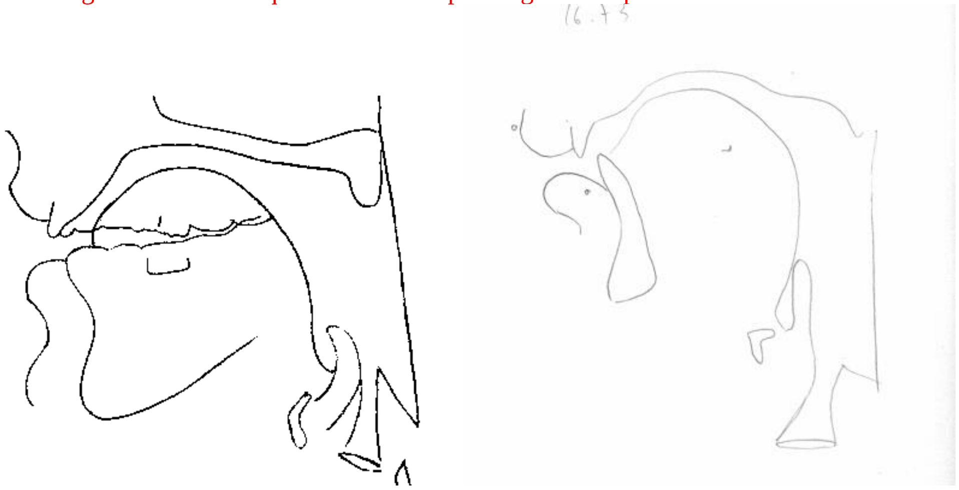

Articulations of vowel /i/ in two different male native speakers of French
(Contours extracted from X Ray data – Institut de Phonétique de Strasbourg – LilPa – Université de Strasbourg)

In our preceding work (Buchaillard et al., 2009), we demonstrated the capacity of the tongue model to generate the phonetically crucial characteristics of the tongue contour and showed which muscles were involved to produce these characteristics. We wrote (p. 2043): "*Qualitatively the tongue shape proposed for /i/ is in good agreement with different kinds of data published in the literature. This is true for the 2D shape in the midsagittal plane, which is consistent with Bothorel et al. (1986) data for French speech sounds. It is also true for the 3D distribution of the*

contacts between the hard palate and the upper dental arch on the one side and the tongue lateral borders on the other side." However, our goal at this time was not to address speaker-specific strategies used to achieve these phonetically crucial characteristics. And in the current study we do focus on these speaker-specific aspects. Comparing the tongue shapes generated for vowel /i/ in the alveo-palatal region in Buchaillard et al. (2009) and in our current study, we see how more accurate our approximation of the speaker-specific tongue contour is now as compared to before. This is the kind of valuable information that we can retrieve from visual assessment.

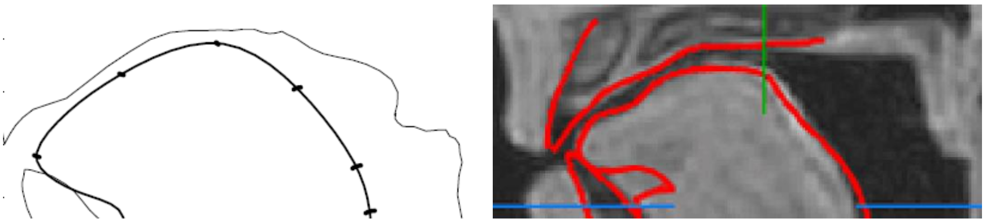

Tongue contour generated for vowel /i/ in Buchaillard et al., (2009) (left panel) and in our current work (right panel).

Lastly, for me, it's not clear why you chose those particular vowels and consonants for your analysis. Edit: This is answered later on. However, I still think that this could be directly mentioned in the results section.

We have modified the text in the Result section, in order to clarify our choice (text page 10, lines 187-191): "We selected four French phonemes: /a/, /u/, /k/, and /t/, in addition to the phoneme /i/. These phonemes were chosen for their specific articulatory characteristics. Indeed, /i/, /a/, and /u/ are the extreme cardinal vowels found across all the world's languages; they are the most distant from each other, offering a maximal covering the vowel space. As for /k/ and /t/, they are both stop consonants: the former is a posterior (velar) consonant, and the latter an anterior (dental) one; they are also representative of very different points of articulation."

References

Alvarez, P., El Mouss, M., Calka, M., Belme, A., Berillon, G., Brige, P., ... & Vialet, A. (2024). Predicting primate tongue morphology based on geometrical skull matching. A first step towards an application on fossil hominins. *PLoS Computational Biology*, *20*(1), e1011808.

Al-Zanoon, N., Cummine, J., Jeffery, C. C., Westover, L., & Aalto, D. (2024). The effect of simulated radiation induced fibrosis on tongue protrusion. *Biomechanics and Modeling in Mechanobiology*, *23*(5), 1649-1660.

Buchaillard, S., Perrier, P., & Payan, Y. (2009). A biomechanical model of cardinal vowel production: Muscle activations and the impact of gravity on tongue positioning. The Journal of the Acoustical Society of America, 126(4), 2033-2051.

Calka, M., Perrier, P., Ohayon, J., Grivot-Boichon, C., Rochette, M., & Payan, Y. (2021). Machine-learning based model order reduction of a biomechanical model of the human tongue. *Computer Methods and Programs in Biomedicine, 198*, 105786.

Calka, M., Picard, M. C., Perrier, P., Rochette, M., & Payan, Y. (2023, October). Automatic generation of patient-specific finite element meshes of the face and oral cavity for orofacial digital twins. In *Computer Methods in Biomechanics and Biomedical Engineering Volume 26, 2023-Issue sup1: 48th congress of the Société de Biomécanique* (Vol. 26, No. sup1, pp. S196-S198).

Gick, B., Allen, B., Roewer-Després, F., & Stavness, I. (2017). Speaking tongues are actively braced. *Journal of Speech, Language, and Hearing Research*, *60*(3), 494-506.

Isazadeh, A. R., Seikaly, H., Westover, L., & Aalto, D. (2024). Algorithmically designed flaps in tongue reconstruction: a feasibility analysis. International Journal of Computer Assisted Radiology and Surgery, 1-12.

Payan, Y., & Perrier, P. (1997). Synthesis of VV sequences with a 2D biomechanical tongue model controlled by the Equilibrium Point Hypothesis. *Speech communication*, *22*(2-3), 185-205.

Perrier, P., Payan, Y., Zandipour, M., & Perkell, J. (2003). Influences of tongue biomechanics on speech movements during the production of velar stop consonants: A modeling study. *The Journal of the Acoustical Society of America*, *114*(3), 1582-1599.

Stavness, I., Gick, B., Derrick, D., & Fels, S. (2012). Biomechanical modeling of English/r/variants. *The Journal of the Acoustical Society of America*, *131*(5), EL355-EL360.

Rakotomalala, T., Perrier, P., & Baraduc, P. (2022). Trajectories predicted by optimal speech motor control using LSTM networks. In *Interspeech 2022-23rd Annual Conference of the International Speech Communication Association* (pp. 630-634). ISCA.
